# *Thymineless death*, at the origin

**DOI:** 10.3389/fmicb.2015.00499

**Published:** 2015-05-19

**Authors:** Elena C. Guzmán, Carmen M. Martín

**Affiliations:** Departamento de Bioquímica Biología Molecular y Genética, Facultad de Ciencias, Universidad de ExtremaduraBadajoz, Spain

**Keywords:** TLD, replication fork, initiation, *oriC*, rifampicin, DSBs, 2D gel DNA

## Abstract

*Thymineless death* (TLD) in bacteria has been a focus of research for decades. Nevertheless, the advances in the last 5 years, with *Escherichia coli* as the model organism, have been outstanding. Independent research groups have presented compelling results that establish that the initiation of chromosome replication under thymine starvation is a key element in the scenario of TLD. Here we review the experimental results linking the initiation of replication to the lethality under thymine starvation and the proposed mechanisms by which TLD occurs. The concept of this relationship was ‘in the air,’ but approaches were not sufficiently developed to demonstrate the crucial role of DNA initiation in TLD. Genome-wide marker frequency analysis and Two Dimensional agarose gel electrophoresis have been critical methods employed to reveal that initiation events and the degradation of the *oriC* region occur during thymine starvation. The relationships between these events and TLD have established them to be the main underlying causes of the lethality under thymine starvation. Furthermore, we summarize additional important findings from the study of different mutant strains, which support the idea that the initiation of chromosomal replication and TLD are connected.

## Introduction

**FIGURE 1:**
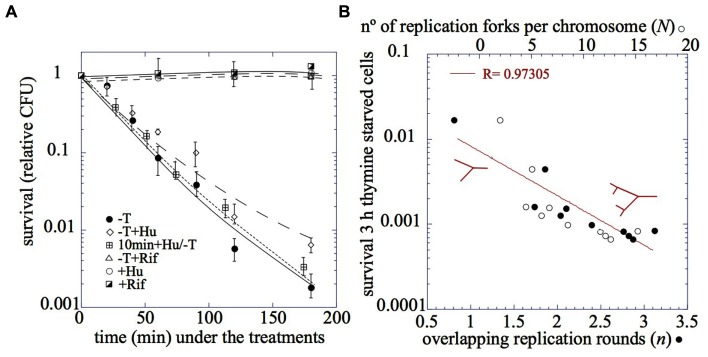
**(A)** The relative cell survival after (●) thymine starvation, (♢) in the presence of hydroxyurea, (△) in the presence of rifampicin, (⊞) 10 min pretreatment with hydoxyurea and after addition of hydroxyurea 75 mM, (○) addition of hydroxyurea 75 mM, (◪) addition of rifampicin. **(B)** The relationship between the number of replication rounds per chromosome, *n*, or the number of replication forks per chromosome, *N,* and the relative viability after 3 h of thymine starvation. The red drawing corresponds to the chromosome configuration with one or two cycles of chromosome replication. (adapted from Martín and Guzmán, 2011; Martín, 2014).

*Thymineless death* (TLD) is defined by the loss of viability that occurs in a culture of a *thyA* defective mutant strain when deprived of thymine (**Figure 1A**). It was first reported by Barner and Cohen 60 years ago (Barner and Cohen, 1954). In the ensuing 60 years a number of other laboratory groups have studied this phenomenon and have attempted to elucidate its mechanism. Throughout the years, TLD has been associated with DNA damage and DNA recombination structures, as well as their outcomes: SOS induction, filamentation, mutagenesis, loss of plasmids, or induction of suicide modules and prophages, among others (Ahmad et al., 1998). However, the relative contribution of these factors, individually or in combination, to TLD remains unknown. A novel and critical aspect has emerged in the last 5 years: the initiation of chromosomal replication. Abortive events in attempted initiation during thymine starvation may be associated with the observed degradation of the *oriC* DNA sequence that eventually leads to TLD.

The goal of this minireview is to detail the experiments and various approaches that establish the initiation of replication as a key element in the continuously evolving story of TLD.

## The Unbalanced Growth Model

The first general model to explain TLD was the recognition of *unbalanced growth* generated under thymine starvation (Barner and Cohen, 1954). Certainly, DNA replication eventually stops in thymine-starved cells, while the processes of DNA recombination and repair may continue (Nakayama et al., 1994), and neither RNA nor protein synthesis are greatly affected (Hanawalt et al., 1961; Maaloe and Hanawalt, 1961; Nakayama and Hanawalt, 1975). Nevertheless, the notion of *unbalanced growth* was too vague, and it was shown that it cannot be the sole cause of TLD. Various experimental conditions suppressed TLD while maintaining an imbalance between DNA and protein synthesis. Examples of these conditions include the starvation of thymine in the presence of chloramphenicol (Okagaki et al., 1960) during which RNA synthesis remains active, or the heat inactivation of the proteins required for chromosomal initiation, DnaA or DnaC, (Bouvier and Sicard, 1975) during which RNA and protein synthesis are not affected. None of these conditions would predict the suppression of TLD according to the *unbalanced growth* model.

## TLD is Related to DNA Replication

Thymine is exclusively incorporated into DNA during the replication process; therefore, TLD has been associated for many years with DNA replication. Early observations described conditions under which TLD was suppressed in cells that had completed replication rounds (Maaloe and Hanawalt, 1961). Additionally, TLD has been shown to be affected differently under inactivation of several replication proteins (Bouvier and Sicard, 1975; Nakayama et al., 1994). Thus, a direct involvement of the chromosomal replication process in TLD is apparent.

### TLD Correlates with the Number of Replication Forks, but they are Not Required to be Fully Active

A relationship between the magnitude of the lethality under thymine starvation and the number of replication forks has been well established. Both parameters were determined in the strain MG1693 *thyA175* grown under various conditions to achieve different numbers of replication rounds per chromosome, *n*, (Sueoka and Yoshikawa, 1965). Survival after thymine starvation (**Figure 1A**) was found to inversely correlate with *n* and the number of forks per cell, *N,* before thymine starvation (**Figure 1B**; Martín and Guzmán, 2011; Martín, 2014). However, it is not clear whether the activity of the replication forks is required for the lethality. TLD is not suppressed when thymine starvation is accomplished under conditions of DNA inhibition, such as the addition of hydroxyurea (Morganroth and Hanawalt, 2006; Kuong and Kuzminov, 2009; Martín and Guzmán, 2011; **Figure 1A**) or the incubation of a *dnaBts* mutant at 42°C (Bouvier and Sicard, 1975). Controversy has arisen because the two approaches used to inactivate the replication forks do not show a clear-cut inhibition of DNA synthesis (Kuong and Kuzminov, 2009, 2010). Nevertheless, the complete absence of effect on TLD either after hydroxyurea addition or incubation of *dnaBts* at 42°C suggests that TLD is not dependent on the level of activity of the replication forks. Furthermore, the observation that the addition of hydroxyurea 10 min before thymine starvation does not change the kinetics of TLD supports this idea (**Figure 1A**; Martín and Guzmán, 2011; Martín, 2014).

### DNA Fragmentation and Recombinant DNA Intermediates are Not Sufficient to Account for TLD

When considering the idea that the replication forks are targeted during thymine starvation, the primary assumption is that TLD results from DNA damage brought about by thymine starvation on its target. What effects could thymine starvation produce on the replication forks? Different models have suggested two primary sources of TLD that are not mutually exclusive: DNA breakage and DNA recombination intermediates, which have been associated either with RNA synthesis during thymine starvation (Nakayama and Hanawalt, 1975) or to different recombination pathways such as RecA/RecBCD or RecFOR (Fonville et al., 2010; Kuong and Kuzminov, 2010).

DNA breakage has been observed under thymine starvation; thus, the occurrence of single-strand breaks (SSBs), DNA single-strand gaps (DNA ss-gaps; Nakayama and Hanawalt, 1975), and double strand breaks (DSBs; Guarino et al., 2007; Kuong and Kuzminov, 2010; Martín and Guzmán, 2011) have been shown to occur during *thymineless* incubation of bacteria. Furthermore, Nakayama et al. (1994) demonstrated the presence of DNA recombination intermediates, known as “non-migrating DNA” (nmDNA), in thymine-starved cells. Nevertheless, Martín and Guzmán (2011) have shown that neither the number of DSBs nor the level of nmDNA correlated with TLD. DSBs appeared in the DNA of thymine-starved cells in the presence of rifampicin, a drug that prevents TLD. Thus, DSBs might be necessary but not sufficient to cause TLD. In addition, DNA recombination intermediates (nmDNA) were not observed under thymine starvation when hydroxyurea was present (Martín and Guzmán, 2011). Because death still occurs under thymine starvation in the presence of hydroxyurea (**Figure 1A**), DNA recombination intermediates may be associated with, but are not essential for, TLD. Thus, what is the critical condition for TLD to occur?

## Initiation of Replication is a Key Element in TLD

Although TLD has been associated for many years with replicating cells, and recent results have demonstrated a correlation between TLD and the number of replication forks, two results have suggested that additional components of the replication process are involved in TLD. First, TLD is suppressed by inhibiting RNA or protein synthesis, or both, as is observed in experimental conditions including the presence of rifampicin (Hanawalt, 1963; Martín and Guzmán, 2011; Martín et al., 2014) or chloramphenicol or in amino acid-starved cells (Martín, 2014). Second, TLD is avoided by inactivating the DnaA protein (Bouvier and Sicard, 1975; Nakayama et al., 1994; Martín et al., 2014). The nexus between these three conditions (RNA and protein synthesis and the active form of DnaA protein) is their requirement at the initiation of replication. The inhibition of initiation by any of these treatments does not affect elongating replication forks, which can progress until the replication of the chromosome is completed. Thus, a correlation has been found between initiation of new replication rounds under thymine starvation and TLD. The causative link between DNA initiation and TLD has been established by several lines of evidence detailed below.

### New Initiations Occur Under Thymine Starvation

The occurrence of new initiation events after restoring thymine to thymine-starved cells was first reported in the 1960s (Pritchard and Lark, 1964) and confirmed within the past 5 years (Martín and Guzmán, 2011; Martín et al., 2014). Furthermore, it has been shown by different approaches that initiation events do occur during thymine starvation.

**FIGURE 2:**
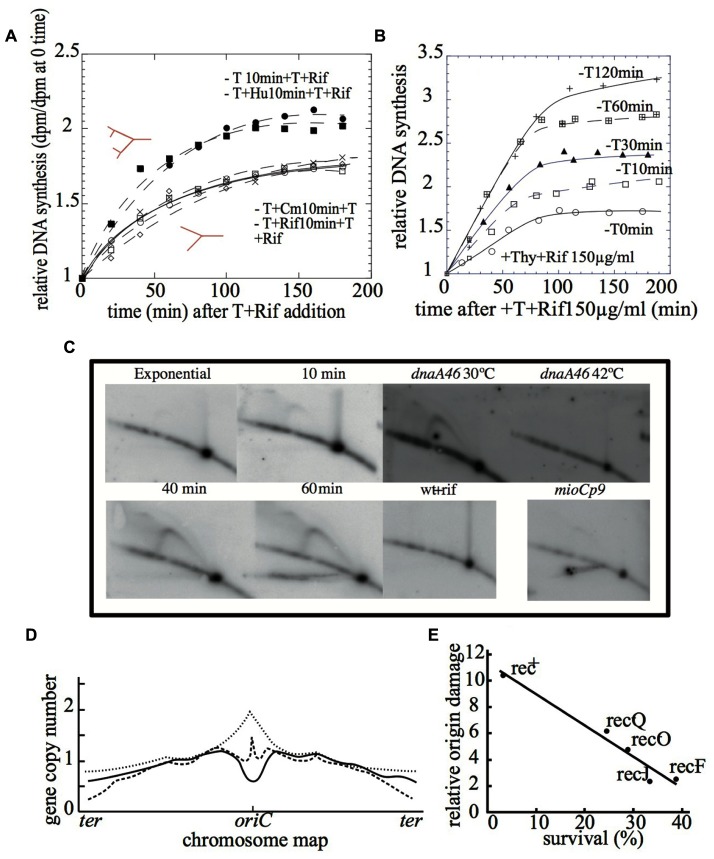
**(A)** The relative DNA accumulation in the presence of rifampicin after thymine restoration to 10 min thymine-starved cells in the presence of rifampicin, chloramphenicol, hydroxyurea, or any drugs (Martín and Guzmán, 2011). **(B)** The relative DNA accumulation in the presence of rifampicin after thymine restoration to cells under thymine starvation for different extents of time (Martín et al., 2014). **(C)** The 2D gel DNA analysis of exponentially growing cells at 0, 10, 40, and 60 min of thymine starvation in the wild type strain; at 60 min and 30 or 42°C in the *dnaA46* thermosensitive mutant; in the presence of rifampicin and in the *mioCp9* mutant (adapted from Martín et al., 2014). **(D)** The Marker Frequency Analysis of the wild type strain (solid line), the *recA* (dotted line) mutant, or the *recBC* mutant (broken line) after 4 h of thymine starvation (adapted from Kuong and Kuzminov, 2012). **(E)** The correlation between the relative *origin* damage and percent of survival after 3 h of thymine starvation in the wild type strain and Rec defective mutants (*recQ, recO, recJ,* and *recF*) (adapted from Sangurdekar et al., 2010).

*(i) Replication runouts* – This approach is based on the *ΔG* and the Δ*G′* values. Briefly, ΔG refers to the relative increase in the amount of DNA after the inhibition of new rounds of chromosome replication, a condition achieved by adding 150 μg ml^-1^ of rifampicin to an exponentially growing culture (Sueoka and Yoshikawa, 1965; Zaritsky and Pritchard, 1971). Δ*G′* represents the relative increment of DNA in a culture subjected to 10 min of thymine starvation followed by restoration of thymine in the presence of 150 μg ml^-1^ of rifampicin to inhibit new initiations. Thus, if new initiations occur in the thymine-starved cells, then the Δ*G′* value is expected to be higher than the Δ*G* value (Jiménez-Sánchez and Guzmán, 1988). The results in **Figure 2A** show that the value of Δ*G′* after the addition of thymine to 10-min thymine-starved cultures is higher than Δ*G*. These results show that new initiations occurred at a number of origins (*i*) under thymine starvation. Initiation events also occurred in the presence of hydroxyurea, but they were suppressed when the 10-min period of thymine starvation was performed in the presence of rifampicin or chloramphenicol. These results show a correlation between chromosomal initiation and lethality under thymine starvation (Martín and Guzmán, 2011; Martín et al., 2014).

Furthermore, it was shown that the number of initiations at *oriC* increased with the amount of treatment time (**Figure 2B**), correlating with a loss of colony-forming units on solid medium (**Figure 1A**). Flow cytometry profiles of the replication runouts after thymine addition to cultures previously exposed to increasing time periods of thymine starvation indicated that only the *thymineless-*initiations that had occurred during the first 30 min could be repaired to allow complete chromosome replications (Martín et al., 2014).

*(ii) Visualization of the oriC replication intermediates under thymine starvation by 2D gels* – The analysis of the replication fork progression at one specific position of the chromosome can be resolved by performing two-dimensional DNA gel electrophoresis (2D gels; Brewer and Fangman, 1987; Schwartzman et al., 2012). The results with 2D gels show the different DNA structures that occur in the *oriC* region during thymine starvation (**Figure 2C**). The bubble arcs detected after 40 min indicate initiation events at *oriC*, while the double-Y structures are most likely produced by two forks encountering each other at *oriC*. These structures indicate that replication rounds are initiated during thymine starvation in the *oriC* region, although the possibility of initiations at different locations cannot be excluded. A simple-Y arc, corresponding to accumulated Y-shaped replication intermediates, was clearly detected after 10 min of thymine starvation. This indicates the arrest of replication forks within the *oriC* region and also strongly suggests that some initiations occur outside of the restriction fragment but still in the vicinity of *oriC*.

The experiments using the mutant strains confirmed the occurrence of initiation during thymine starvation. Consistent with the suppression of TLD, none of the DNA intermediates observed in wild type strains under TLD conditions were detected by 2D gels when initiations were inhibited by rifampicin or by DnaA inactivation (**Figure 2C**; Martín et al., 2014).

*(iii) Copy number of oriC sequences increases during first 30 min under thymine starvation* – The third approach providing evidence that new initiations occur during thymine starvation has been the quantification of the *ori/ter* ratio by performing either quantitative PCR (Sangurdekar et al., 2010) or Marker Frequency Analysis (dot-blot hybridization; Kuong and Kuzminov, 2012). The frequency of a gene along the chromosome, *f(a)*, follows a function that depends upon the number of replication rounds per chromosome, *n*, and the position of the gene relative to the origin of replication, *x*, being *f(a)* = 2*^n^*^(1-^*^x)^* (Sueoka and Yoshikawa, 1965).

Determination of the *ori/ter* ratio after 30 min of thymine starvation yielded a value higher than that obtained when the cells were growing exponentially (Kuong and Kuzminov, 2012). The overall results show that *thymineless*-initiation events do occur. With the passage of time, the number of origins decreases, revealing a progressive loss of DNA in the *oriC* sequence (Sangurdekar et al., 2010; Kuong and Kuzminov, 2012) as detailed below.

### *oriC* is Degraded Under Thymine Starvation in a Rec-Dependent Manner

Marker Frequency Analysis on the scale of the whole chromosome by gene arrays is becoming the standard method of analyzing the replication pattern in bacteria. The results of the comparative genomic hybridization of chromosomal DNA after 3–4 h of thymine starvation revealed the loss of the *oriC* region (Sangurdekar et al., 2010; Kuong and Kuzminov, 2012; **Figure 2D**). Furthermore, this conclusion is supported by the loss of *oriC*-containing foci during TLD, which was revealed by fluorescence *in situ* hybridization (FISH; Fonville et al., 2010). An explanation for these observations could rely upon the degradation of the *oriC* region originated from DSBs nearby. An interesting question arises at this point: why and how is the *oriC* sequence preferentially degraded? One of the better-known triggers of chromosomal fragmentation is the incorporation of uracil into DNA. This occurs *dut* mutants, in such a way that the chromosome fragmentation exhibits a gradient that parallels the replication gradient (Kouzminova and Kuzminov, 2008). Thus, it may be possible that the distinctive degradation of the *oriC* region is explained by the AT-rich feature of the *oriC* sequence, which could allow for a very significant incorporation of uracil in the absence of thymine.

Several defective repair/recombination mutant strains have been assayed for DNA damage impacting the *oriC* region (Sangurdekar et al., 2010). These include those affecting enzymes that process and repair single-stranded gaps such a *recFOR, recJ,* or *recQ*. All of them are, to some extent, resistant to TLD and contain quantitatively lower origin DNA damage than in wild type strains (**Figure 2E**), thus connecting the extent of DNA damage at the *oriC* region to the lethality under thymine starvation. Interestingly, *recA* defective mutant starved for thymine for 4 h displayed an *ori/ter* ratio even higher than the initial value before treatment (Kuong and Kuzminov, 2012; **Figure 2D**). RecA is a protein that plays a central role in homologous DNA recombination and repair. Nevertheless, it has been reported that either it is not required for TLD (Nakayama et al., 1982) or it may partially palliate it (Fonville et al., 2010; Kuong and Kuzminov, 2010). Thus, the reported absence of *oriC* degradation in a *recA* mutant during thymine starvation would support the alleviated TLD observed in the *recA* mutant (Kuong and Kuzminov, 2012), consistent with the idea that TLD would depend on the origin degradation triggered by RecA-promoted recombinational misrepair.

The provocative exception is the feature exhibited by a *recBC* defective mutant, which has been described to be hypersensitive to TLD, although no *oriC* degradation is observed in this genetic background (Kuong and Kuzminov, 2010, 2012). The RecBCD complex has both helicase and exonuclease activities, and it initiates the repair of DSBs by homologous recombination in combination with RecA protein (Michel et al., 2007; Dillingham and Kowalczykowski, 2008). Because *recBC* mutant cells do not degrade double-strand DNA ends and cannot repair DSBs, the TLD in this defective mutant likely could be related to its inability to repair DSBs at the original replication forks, even though the *oriC* region is not degraded (**Figure 2D**).

### The Transcription-Dependent Step of Initiation is the Target for Rifampicin Suppression of TLD

*Thymineless death* suppression by rifampicin was observed in early studies (Nakayama and Hanawalt, 1975), but its mechanism of action has not yet been elucidated. This problem has been analyzed by two different approaches.

*(i) The effects of different concentrations of rifampicin* – It has been shown that the activity of the RNA polymerase in thymine-starved cells modulates both the initiation of DNA replication under thymine starvation and TLD (Martín et al., 2014). Interestingly, suppression of TLD by rifampicin in a *ΔdatA* mutant was achieved when treated with 1,000 μg ml^-1^ of the drug but not with 150 μg ml^-1^ (Martín and Guzmán, 2011). It has been suggested that in a *ΔdatA* strain, less RNA transcription around *oriC* would be required for initiation to occur because more DnaA protein would be available to open the *oriC* sequence during initiation in this mutant (Morigen et al., 2005). Accordingly, the *ΔdatA* strain initiates chromosome replication in the presence of 150 μg ml^-1^ rifampicin, explaining the observed TLD, while addition at 1,000 μg ml^-1^ completely inhibits the transcription requirement for initiation, hence suppressing TLD.

*(ii) mioC and gid defective mutant strains* – The importance of *mioC* and *gid* gene transcription for the initiation of chromosomal replication at *oriC* is widely accepted (Messer, 1972; Baker and Kornberg, 1988). Nevertheless, neither the deletion of *PmioC112* or *Pgid113* promoters, nor the constitutive transcription from the *mioCp9* promoter have a large effect on the cell cycle. However, reduction in initiation efficiency has been observed in rich medium (Bates et al., 1997; Molina et al., 1999; Su’etsugu et al., 2003). Under thymine starvation, altered *mioC* and *gid* transcription limited the initiation process and TLD was alleviated (Martín et al., 2014). The relevance of these results relies on the fact that TLD alleviation in these mutants must be related to the alteration of the normal transcription levels around *oriC*. This effect on TLD, combined with the broad reduction in chromosome initiation intermediates in these mutants (**Figure 2C**), strongly suggests that the transcription-dependent step of the initiation is the target for rifampicin suppression of TLD, hence being critical for TLD.

## Conclusion

Overall, these experimental approaches pinpoint the initiations at the *oriC* region as the main targets for TLD in wild type strains. Thus, if DNA initiation is allowed under thymine starvation, death occurs likely due to the lethal consequences of the presence of DSBs, DNAss gaps, and DNA recombination intermediates at the *origin* that eventually result in *oriC* region degradation. If initiation is inhibited (*dnaA46* mutant, rifampicin, chloramphenicol) or impaired (*mioC, gid, ΔdatA*, sub-inhibitory rifampicin concentrations), TLD is subsequently suppressed or alleviated, respectively. Thus, the observed correlation between TLD and the number of replication forks could reflect not only the importance of the forks as targets, but also the quantitative relationship between TLD, and the number of *origins* per chromosome, 2^*n*^.

Regarding *thymineless*-initiation events in *thyA* mutants that are otherwise wild type cells, the observations could be divided into two stages. During the first 30–60 min following thymine starvation the instability of DNA ss-gaps and the resulting DNA degradation behind the replication forks (and/or different source; Kuong and Kuzminov, 2012), might provide the dNTPs necessary to support new initiations, residual DNA elongation and the repair of the replication forks. According to the results from different authors, initiation events (supposed to occur at the first stage) are required to yield TLD, as lethality has not been observed in thymine-starved cells under conditions inhibiting DNA initiation.

The second stage would proceed after 30–60 min when the new thymineless-initiation events would generate unrepaired DSBs or DNAss gaps together with unresolved DNA recombination intermediaries at the origin, somehow triggering the unique degradation of the *oriC* region that acts as the major lethal effect of thymine starvation. Supporting this explanation, it also has been shown that the extent of *origin* degradation (supposed to occur at the second stage) correlates with the magnitude of TLD (Sangurdekar et al., 2010). At this point, the role of the recombination/repair enzymes reflects the different sensitivities to thymine starvation described above. Accordingly, several independent pathways accounting for TLD has been proposed (Courcelle, 2005; Nakayama, 2005; Kuong and Kuzminov, 2010) and also supported by Rosenberg’s lab (Fonville et al., 2010, 2011; Hamilton et al., 2013).

Several questions arise from this tentative model. The first one is whether new DNA initiation is a requisite for origin degradation. Second, what is the mechanism by which the *oriC* region is selectively degraded? Third, according to this proposal the DSBs located outside the origin region do not seem to be lethal or, alternatively, the inhibition of DNA initiation is counteracting their potentially lethal effect. Therefore, what is the mechanism of that phenomenon?

The advances in knowledge about TLD mechanisms have been impressive in the past 5 years. New technologies and approaches have evolved to provide novel insights, but TLD is still like a black hole – you know how you got into it but you never know where you will end up as time passes.

## Conflict of Interest Statement

The authors declare that the research was conducted in the absence of any commercial or financial relationships that could be construed as a potential conflict of interest.

## References

[B1] Ahmad S. I. Kirk S. H. Eisenstark A. (1998). Thymine metabolism and thymineless death in prokaryotes and eukaryotes. *Annu. Rev. Microbiol.* 52 591–625. 10.1146/annurev.micro.52.1.591 9891809

[B2] Baker T. A. Kornberg A. (1988). Transcriptional activation of initiation of replication from the E. *coli* chromosomal origin: an RNA-DNA hybrid near *oriC*. *Cell* 55 113–123. 10.1016/0092-8674(88)90014-1 2458841

[B3] Barner H. D. Cohen S. S. (1954). The induction of thymine synthesis by T2 infection of a thymine requiring mutant of *Escherichia coli*. *J. Bacteriol.* 68 80–88. 13183905 10.1128/jb.68.1.80-88.1954 PMC357338

[B4] Bates D. Boye E. Asai T. Kogoma T. (1997). The absence of effect of *gid* or *mioC* transcription on the initiation of chromosomal replication in *Escherichia coli*. *Proc. Natl. Acad. Sci. U.S.A.* 94 12497–12502. 10.1073/pnas.94.23.12497 9356478 PMC25015

[B5] Bouvier F. Sicard N. (1975). Interference of *dna-ts* mutations of *Escherichia coli* with *thymineless death*. *J. Bacteriol.* 124 1198–1204. 1104577 10.1128/jb.124.3.1198-1204.1975 PMC236027

[B6] Brewer B. J. Fangman W. L. (1987). The localization of replication origins on ARS plasmids in *S. cerevisiae*. *Cell* 51 463–471. 10.1016/0092-8674(87)90642-8 2822257

[B7] Courcelle J. (2005). Recs preventing wrecks. *Mutant. Res.* 557 217–227. 10.1016/j.mrfmmm.2005.03.019 16011837

[B8] Dillingham M. S. Kowalczykowski S. C. (2008). RecBCD enzyme and the repair of Double-Stranded DNA Breaks. *Microbiol. Mol. Biol. Rev.* 72 642–671. 10.1128/MMBR.00020-08 19052323 PMC2593567

[B9] Fonville N. C. Bates D. Hastings P. J. Hanawalt P. C. Rosenberg S. M. (2010). Role of RecA and the SOS response in thymineless death in *Escherichia coli*. *PLoS Genet.* 6:e1000865 10.1371/journal.pgen.1000865 PMC2832678 20221259

[B10] Fonville N. Vaksman Z. DeNapoli J. Hastings P. J. Rosenberg S. M. (2011). Pathways of resistance to thymineless death in *Escherichia coli* and the function of UvrD. *Genetics* 189 23–36. 10.1534/genetics.111.130161 21705756 PMC3176125

[B11] Guarino E. Salguero I. Jiménez-Sánchez A. Guzmán E. C. (2007). Double-strand break generation under deoxyribonucleotide starvation in *Escherichia coli*. *J. Bacteriol.* 189 5782–5786. 10.1128/JB.00411-07 17526701 PMC1951825

[B12] Hamilton H. M Wilson R. Blythe M. Nehring R. B. Fonville N. C. Louis E. J. (2013). Thymineless death is inhibited by CsrA in Escherichia coli lacking the SOS response. *DNA Repair (Amst)* 12 993–999. 10.1016/j.dnarep.2013.08.011 24075571 PMC3898814

[B13] Hanawalt P. C. (1963). Involvement of synthesis of RNA in *thymineless death*. *Nature* 198 286 10.1038/198286a0 13952474

[B14] Hanawalt P. C. Maaløe O. Cummings D. J. Schaechter M. (1961). The normal DNA replication cycle. II. *J. Mol. Biol.* 3 156–165. 10.1016/S0022-2836(61)80042-9 13711156

[B15] Jiménez-Sánchez A. Guzmán E. C. (1988). Direct procedure for the determination of the number of replication forks and the reinitiation fraction in bacteria. *Comput. Appl. Biosci.* 4 431–433. 3208175 10.1093/bioinformatics/4.4.431

[B16] Kouzminova E. A. Kuzminov A. (2008). Patterns of chromosomal fragmentation due to uracil-DNA incorporation reveal a novel mechanism of replication-dependent double-stranded breaks. *Mol. Microbiol.* 68 202–215. 10.1111/j.1365-2958.2008.06149.x 18312272

[B17] Kuong K. J. Kuzminov A. (2009). Cyanide, peroxide and nitric oxide formation in solutions of hydroxyurea causes cellular toxicity and may contribute to its therapeutic potency. *J. Mol. Biol.* 390 845–862. 10.1016/j.jmb.2009.05.038 19467244 PMC2728359

[B18] Kuong K. J. Kuzminov A. (2010). Stalled replication fork repair and misrepair during thymineless death in *Escherichia coli*. *Genes Cells* 15 619–634. 10.1111/j.1365-2443.2010.01405.x 20465561 PMC3965187

[B19] Kuong K. J. Kuzminov A. (2012). Disintegration of nascent replication bubbles during thymine starvation triggers RecA- and RecBCD-dependent replication origin destruction. *J. Biol. Chem.* 287 23958–23970. 10.1074/jbc.M112.359687 22621921 PMC3390671

[B20] Maaloe O. Hanawalt P. C. (1961). Thymine deficiency and the normal DNA replication cycle. *I. J. Mol. Biol.* 3 144–155. 10.1016/S0022-2836(61)80041-7 13764647

[B21] Martín C. M. (2014). *Role of DNA Replication Initiation on the Lethality Caused by Thymine Starvation*. Ph.D. thesis, Universidad de Extremadura Badajoz.

[B22] Martín C. M. Guzmán E. C. (2011). DNA replication initiation as a key element in thymineless death. *DNA Repair (Amst.)* 10 94–101. 10.1016/j.dnarep.2010.10.005 21074501

[B23] Martín C. M. Viguera E. Guzmán E. (2014). Rifampicin suppresses thymineless death by blocking the transcription-dependent step of chromosome initiation. *DNA Repair* 18 10–71. 10.1016/j.dnarep.2014.03.004 24742961

[B24] Messer W. (1972). Initiation of deoxyribonucleic acid replication in *Escherichia coli* B-r: chronology of events and transcriptional control of initiation. *J. Bacteriol.* 112 7–12. 4562418 10.1128/jb.112.1.7-12.1972 PMC251374

[B25] Michel B. Boubakri H Baharoglu Z LeMasson M Lestini R. (2007). Recombination proteins and rescue of arrested replication forks. *DNA Repair* 6 967–980. 10.1016/j.dnarep.2007.02.016 17395553

[B26] Molina F. Jiménez-Sánchez A. Zyskind J. W. Guzmán E. C. (1999). Chromosomal insertions localized around *oriC* affect the cell cycle in *Escherichia coli*. *Biochimie* 81 811–818. 10.1016/S0300-9084(99)00216-3 10572293

[B27] Morganroth P. A. Hanawalt P. C. (2006). Role of DNA replication and repair in thymineless death in *Escherichia coli*. *J. Bacteriol.* 188 5286–5288. 10.1128/JB.00543-06 16816201 PMC1539979

[B28] Morigen Molina F. Skarstad K. (2005). Deletion of the *datA* site does not affect once-per-cell-cycle timing but induces rifampin-resistant replication. *J. Bacteriol.* 187 3913–3920. 10.1128/JB.187.12.3913-3920.2005 15939703 PMC1151742

[B29] Nakayama H. (2005). Escherichia coli RecQ helicase: a player in *thymineless death*. *Mutat. Res.* 577 228–236. 10.1016/j.mrfmmm.2005.02.015 15922367

[B30] Nakayama H. Hanawalt P. C. (1975). Sedimentation analysis of deoxyribonucleic acid from thymine-starved *Escherichia coli*. *J. Bacteriol.* 121 537–547. 1090581 10.1128/jb.121.2.537-547.1975 PMC245964

[B31] Nakayama H. Nakayama K. Nakayama R. Nakayama Y. (1982). Recombination-deficient mutations and thymineless death in *Escherichia coli*: reciprocal effects of *recBC* and *recF* and indifference of *recA* mutations. *Can. J. Microbiol.* 28 425–430. 10.1139/m82-064 7046891

[B32] Nakayama K. Kusano K. Irino N. Nakayama H. (1994). Thymine starvation-induced structural changes in *Escherichia coli* DNA. Detection by pulsed field gel electrophoresis and evidence for involvement of homologous recombination. *J. Mol. Biol.* 243 611–620. 10.1016/0022-2836(94)90036-1 7966286

[B33] Okagaki H. Tsubota Y. Sibataini A. (1960). Unbalanced growth and bacterial death in thymine-deficient and ultraviolet irradiated *Escherichia coli*. *J. Bacteriol.* 80 762–771. 13730571 10.1128/jb.80.6.762-771.1960 PMC278928

[B34] Pritchard R. H. Lark K. G. (1964). Induction of replication by thymine starvation at the chromosome origin in *Escherichia coli* *J. Mol. Biol.* 9 288–307. 10.1016/S0022-2836(64)80208-4 14202267

[B35] Sangurdekar D. P. Hamann B. L. Smirnov D. Srienc F. Hanawalt P. C. Khodursky A. B. (2010). Thymineless death is associated with loss of essential genetic information from the replication origin. *Mol. Microbiol.* 75 1455–1467. 10.1111/j.1365-2958.2010.07072.x 20132444

[B36] Schwartzman J. B. Martínez-Robles M. L. López V. Hernández P. Krimer D. B. (2012). 2D gel and their third-dimensional potential. *Methods* 57 170–178. 10.1016/j.ymeth.2012.03.013 22465282

[B37] Sueoka N. Yoshikawa H. (1965). The chromosome of Bacillus subtilis. I. Theory of marker frequency analysis. *Genetics* 52 747–757. 4953222 10.1093/genetics/52.4.747 PMC1210937

[B38] Su’etsugu M. Emoto A. Fujimitsu K. Keyamura K. Katayama T. (2003). Transcriptional control for initiation of chromosomal replication in *Escherichia coli*: fluctuation of the level of origin transcription ensures timely initiation. *Genes Cells* 8 731–745. 10.1046/j.1365-2443.2003.00671.x 12940821

[B39] Zaritsky A. Pritchard R. (1971). Replication time of the chromosome in *thymineles* mutants of *Escherichia coli*. *J. Mol. Biol.* 60 65–74. 10.1016/0022-2836(71)90447-5 4937194

